# Optimizing clarification processes in biopharmaceutical manufacturing through quality by design: Strategies, implications, and future prospects

**DOI:** 10.1002/btpr.70063

**Published:** 2025-08-14

**Authors:** Kyeong‐won Yeop, Hyun‐ju Nam, Chang‐jae Shim, So‐mi Yang, Hyo‐won Kim, Cheon Ik Park, Subhasis Banerjee, Yanglin Mok

**Affiliations:** ^1^ Technical Scientific Solutions (TSS) group, Downstream process, Manufacturing Science and Technology Life Science, Process Solutions Merck Ltd. (An Affiliate of Merck KGaA) Darmstadt, Germany Seoul South Korea; ^2^ Life Science, Process Solutions Merck Life Sciences India Pvt. Ltd. (An Affiliate of Merck KGaA, Darmstadt, Germany) Bangalore India; ^3^ Life Science, Process Solutions Merck Pte Ltd. (An Affiliate of Merck KGaA, Darmstadt, Germany) Singapore Singapore

**Keywords:** clarification, CPP (critical process parameter), CQA (critical quality attribute), depth filter, flux (LMH), harvest, HCP, QbD (quality by design)

## Abstract

Biopharmaceutical manufacturing processes in which the product of interest is extracellularly expressed typically employ a clarification step following cell culture or fermentation. During clarification, crude cell culture fluid or fermentation broth is processed to remove insoluble solids, cells, debris, and other particulates, with the extracellular product of interest retained in the filtrate. Soluble impurities, such as host cell proteins (HCPs), may also be partially removed. Historically, the clarification process has been considered a limited contributor to Critical Quality Attributes (CQA). As part of upstream harvest, many biopharmaceutical companies have not fully developed quality control strategies from process development to manufacturing, complicating the application of Quality by Design (QbD) principles to this step. However, advancements in upstream and downstream processing (DSP) technologies, alongside increasing cell counts and titers, necessitate reevaluating clarification as a critical process contributing to drug product quality. Conducting controlled studies to define the process and establish parameters using QbD principles can improve control over process impurities and facilitate a logical quality control strategy, integrating quality into the process. This article describes a systematic approach to QbD for a harvest clarification process where the product of interest is extracellular and impurities are removed in the filtrate post‐clarification. It highlights methods for optimizing the clarification unit operation using QbD principles, ensuring better process efficiency, and product quality.

## 1 QbD CONCEPT IN BIOPHARMACEUTICAL MANUFACTURING

### 1.1 History of QbD activities

Quality by design (QbD) traces its origins back to the early 1970s, conceptualized by Dr. Joseph M. Juran. In his Quality Handbook (1992),^1^ Dr. Juran outlined the principles of quality planning, emphasizing that the quality of a product could be characterized by two main aspects: “features of the product that meet customer satisfaction” and “freedom from deficiencies.”^1^

Since 1970, numerous experts have been refining products based on this theory. In a more recent contribution from 2014, Jyotsna Balasaheb Jadhav^2^ concisely articulated this concept stating, “Quality by Design means designing and developing manufacturing processes during the product development stage to consistently ensure a predefined quality at the end of the manufacturing process.”^2^

Prior to Jadhav's remark, QbD was defined as “A systematic approach to development that begins with predefined objectives and emphasizes product and process understanding and process control, based on sound science and quality risk management,” as per ICH Q8(R2).^3^

### 1.2 Perspectives from regulatory agencies on the QbD concept

The United States Food and Drug Administration (USFDA) established the Current Good Manufacturing Practices (cGMP) system in 2003 and began focusing on risk assessments for quality control in 2005. Similarly, the European Medicines Agency (EMA) started considering cGMP in 2005, as indicated in their press release titled “The European Medicines Agency Road Map to 2010: Preparing the Ground for the Future.”^4^ Prompted by these initiatives, the International Council for Harmonization of Technical Requirements for Pharmaceuticals for Human Use (ICH) issued guidelines for pharmaceutical development and manufacturing starting in 2005. Since the inception of these activities, the QbD concept has been applied to pharmaceuticals, and the ICH guidelines continue to recommend quality control through QbD.

### 1.3 QbD concept in biopharmaceutical manufacturing

In 2009, Rathore published,^5^ “While QbD has seen more extensive application in the manufacturing of small molecules, such as employing the Process Analytical Technology (PAT) concept in crystallization or freeze‐drying processes for parenteral drugs, the biopharmaceutical sector's literature on the application of QbD remains limited. Cook et al.^6^ presented a case study that used design of experiments to identify key and critical process parameters and their targets for a hydrophobic interaction chromatography process in monoclonal antibody (mAb) purification.”^7^ Initially, QbD was implemented in the production of small molecule generics, but since 2009, the biopharmaceutical industry has increasingly focused on adopting QbD principles. However, Qbd is still not mandatory for a regulatory submission of a drug.

Over the past decade, the biotechnology industry and regulators, particularly the FDA, have established the foundation for QbD implementation, overcoming various challenges that could impede its success. As a result, QbD tools and methodologies are increasingly becoming integrated into the workflows of pharmaceutical manufacturers worldwide.^7^

The evolution of QbD in biopharmaceuticals signifies a pivotal shift in drug development and manufacturing, influenced by key regulatory milestones and a movement toward more structured, science‐driven strategies. Beginning in the late 1990s with the FDA's redirection of focus and the enactment of the FDA Modernization Act in 1997, the pharmaceutical industry embarked on a new era. This evolution continued with the Pharmaceutical Current Good Manufacturing Practices (cGMP) for the 21st Century initiative in 2002, further established by the release of ICH Q8, Q10, and Q11 guidelines. These developments laid the foundation for incorporating QbD principles into biopharmaceutical processes, emphasizing the importance of thoroughly understanding product and process design through scientific knowledge and risk management, thereby improving product quality and manufacturing efficiency.^8^

### 1.4 QbD concept for mAb products

**FIGURE 1:**
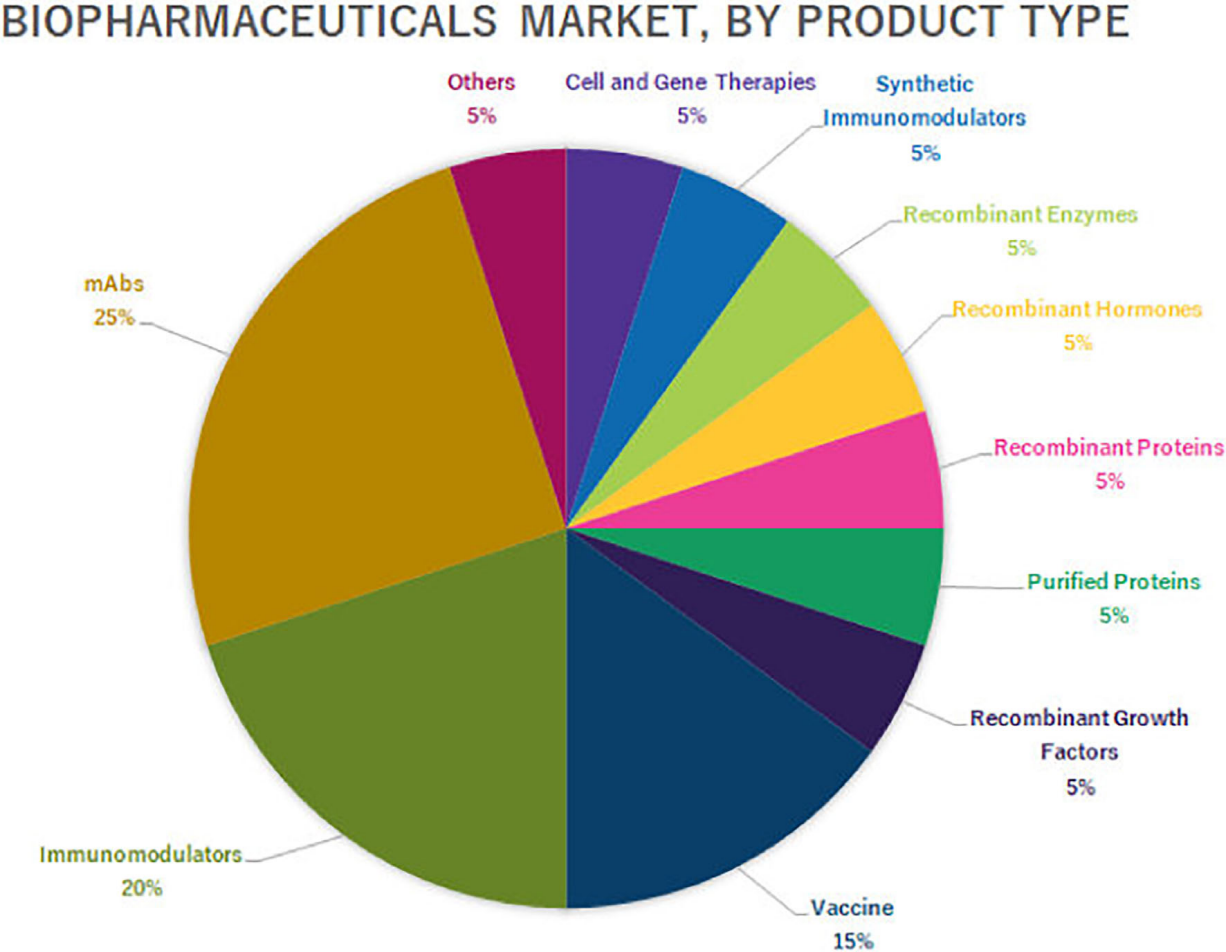
Biopharmaceuticals market, by product type.

The concept of QbD has been applied to the biopharmaceutical industry since 2009, initially advocated by regulatory authorities such as the US FDA and the EMEA to enhance cGMP. The development and approval of mAb products have shown a consistent increase from 2009 to 2021, with about 10 antibodies approved in 2009, then about 50 antibodies approved in 2021.^9^ In Figure 1, the market trends for various biopharmaceutical product types, as reported in Biopharmaceutical Market Share Reports, are shown.^10^, ^11^, ^12^, ^13^, ^14^, ^15^, ^16^ In Figure 1, we infer that monoclonal antibodies represent a large fraction of the biopharmaceutical product types listed.

Numerous case studies on mAbs have focused on aspects like the absence of major or unexpected degradation products and the identification of degradation pathways to inform control strategies, highlight the application of QbD principles.^17^

In line with this trend and the evolving QbD landscape, the WHO published guidelines specifically for mAb products in April 2022. These guidelines outline the purpose, scope, and terminology, emphasizing: Providing regulatory guidance and considerations for marketing authorization that encompass mAb. Covering mAbs of all isotypes and related proteins based on antibody frameworks. Furthermore, the guidelines encompass Manufacturing and Quality Control and guidance for National Regulatory Authorities.

### 1.5 Importance of QbD on biopharmaceuticals

Reflecting on the history and concept of QbD for mAb products, it is evident that QbD is well integrated into the industry, proving essential for quality control such that quality is embedded into the process rather than inspecting quality in the final product. The quality of biopharmaceuticals directly impacts patient safety, necessitating stringent control. The QbD concept, recommending a systematic approach to manufacturing and process development, is crucial not only for patient safety but also for manufacturers, facilitating safe and efficient production with robust control over their processes.

## 2 GENERAL PURIFICATION PROCESS OF BIOPHARMACEUTICALS AND CLARIFICATION STRATEGIES

### 2.1 General overview of clarification

In biopharmaceutical processes, clarification refers to the cell culture harvest step during the upstream where the crude cell culture broth or fermentation broth is separated to remove insoluble solids, cells, cell debris, and other particulate matter from the desired product solution. This step is crucial for preparing the feedstock for subsequent downstream purification processes.

**FIGURE 2:**
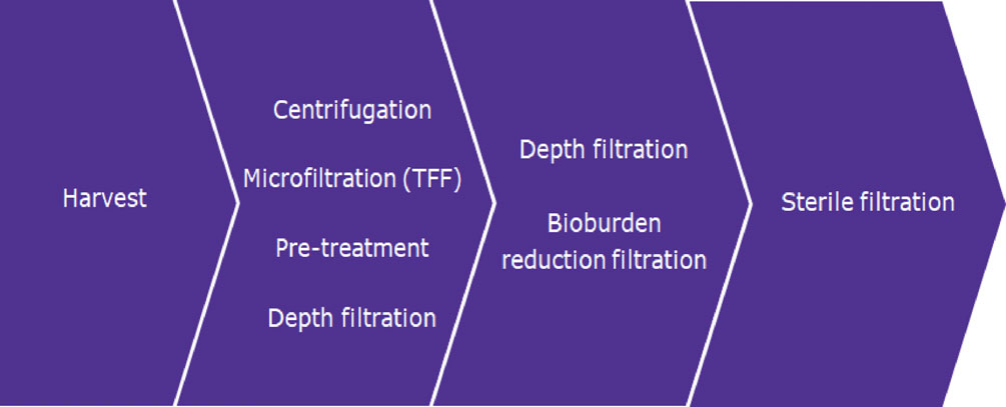
Options for primary and secondary clarification steps in the upstream biomanufacturing process.

The clarification process typically can involve various unit operations as options such as centrifugation,^18^ filtration,^19^ and/or sedimentation by means of pretreatment (e.g., salts,^20^ acids,^21^ and polymers^22^, ^23^, ^24^). In order to achieve a feedstream with sufficient clarity for a downstream purification process, either one or a combination of the techniques can contribute to an efficient clarification of the feedstream (Figure 2). These methods help to separate the solid components from the liquid phase. A recent trend within the biopharmaceutical industry has been toward the adoption of single‐use encapsulated depth filtration technologies, which bring strategic advantages in terms of cost effectiveness, operating efficiency, and faster product development^25^

In the recent years, as cell densities and product titers have risen in upstream to meet the growing demand for biopharmaceuticals, pre‐treatment approaches based on precipitation or flocculation have been investigated as alternative technologies for the efficient removal of particulate solids and soluble impurities in order to ease burdens on downstream purification steps. Precipitation methods can reduce the solubility of undesired solutes, leading to the formation of solid particles, while flocculants can destabilize bio‐colloidal suspensions by promoting the aggregation of dispersed particulates into larger clusters, thereby increasing the average particle size distribution. Due to the increased particle size due to pre‐treatment, most pre‐treatment methods are recommended to couple with depth filters which have large pore ratings and capacities. Singh et al.^26^ compared commonly used precipitants and flocculants in the clarification of therapeutic biologics (Table 1, ^21^, ^23^, ^24^, ^27^, ^28^, ^29^, ^30^, ^31^, ^32^, ^33^, ^34^, ^35^, ^36^, ^37^, ^38^, ^39^, ^40^, ^41^, ^42^, ^43^, ^44^, ^45^, ^46^, ^47^, ^48^, ^49^, ^50^, ^51^, ^52^, ^53^). While modes of action can vary depending on the chemicals or polymers used, both of these approaches can improve the productivity and efficiency of the downstream purification process. Although pre‐treatment is a robust method to enable the effective reduction of impurities, there are several implementation challenges to address. These include the cost‐effectiveness, robustness, and suitability in the process, as well as the validation of the removal of flocculation reagents and the validation of subsequent clearance in the downstream process. These factors should be considered prior to the incorporation of flocculation and precipitation technologies in the clarification process.

**TABLE 1:** Comparison of precipitants and flocculants commonly used in pre‐treatment processes.

Pre‐treatment Method	Precipitants/flocculants	Mode of action	Operation pH	Dosage window	Yield (%)	Impurity reduction	Technical challenges	References
Precipitation	Low pH‐metals	Charge neutralization	4.5–5.5	N/A	>85	HCP: <1 LRV DNA: 1–3 LRV HMW: none viruses: NT	Yield loss, scalability, product stability	21, 27, 28, 29, 30
	Organic solvents	Solubility reduction	6.5–7.5	10%–40% (w/v)	>85	HCP:<1 LRV DNA:<1 LRV HMW: none viruses: NT	Prolonged stirring, product stability, hazardous waste	31, 32
	PEG‐metals	Steric exclusion hydrophobic interaction	5.5–8.5	5–15% (w/v)	>80	HCP: <1 LRV DNA:<3 LRV HMW: yes viruses: NT	Selectivity, residual clearance	33, 34, 35, 36
	Caprylic acid	Coagulation and charge neutralization	4.5–5.5	0.1%–10% (w/v)	>90	HCP: 1–3 LRV DMA: 3–5 LRV HMW: none viruses: >3LRV	Yield loss, solid liquid separation, scalability	37, 38, 39
	Affinity (e.g., ELPs)	Affinity stimulus response	6.5–7.5	ELP to Product ratio of 4:1	>90	HCP: 1–3 LRV DMA: 3–5 LRV HMW: none viruses: NT	High cost, toxicity concerns	40, 41, 42, 43
Anionic flocculation	PVS PAA CMD	Charge interaction with the product	6.5–7.5	10–60 (pg/TCD)	>90	HCP: <1 LRV DNA: 3–5 LRV HMW: none viruses: NT	Product stability, toxicity concerns	44, 45, 46
Cationic flocculation	pDADMAC PEI polyamino acid	Electrostatic hydrogen bonding	4.5–7.5	10–60 (pg/TCD)	>90	HCP:<1 LRV DNA: 4–6 LRV HMW: none viruses: NT	Scalability, variability, toxicity concerns	23, 24, 47, 48, 49
Mix‐mode flocculation	AMPS‐AB2 copolymer modified benzyl poly(allylamine)	Electrostatic hydrophobic hydrogen bonding	4.5–7.5	10–500 (pg/TCD)	>90	HCP: <1 LRV DNA: 3–5 LRV HMW: yes viruses: >3LRV	Product stability, toxicity concern	50, 51, 52, 53

With high titers in mAb upstream, the binding capacity of protein A and ion exchange (IEX) chromatography resins has remained limited, and this poses a significant challenge for traditional purification processes to remove additional impurities.^54^, ^55^, ^56^ For example, production‐scale chromatography columns typically have diameters up to two meters, and it is technically and economically challenging to introduce larger columns into existing biopharmaceutical production facilities.^57^ Consequently, alternative methods have been sought, including the adoption of precipitation techniques from other industries in the biopharmaceutical sector.^58^, ^59^ By adjusting pH and adding flocculants to the harvest sample, impurities can be induced to aggregate, which is removed by the subsequent filtration unit operation, with the mAb product remaining in the supernatant. This process facilitates solid/liquid separation in the clarification process. Acids and charged polymers have been commonly used as flocculants.^37^, ^60^, ^61^, ^62^, ^63^, ^64^

Adjusting pH with acid is operationally simple and does not require complicated analytical methods to detect residuals. However, targeting an appropriate pH is critical, as it can impact the stability of the target product, especially if the product demonstrates a poor stability at low pH conditions.^37^, ^63^ The addition of charged polymers is quite effective at inducing the aggregation of particle solids and soluble impurities; however, to achieve the same flocculation results at varying process scales, a scalability study may be required. Furthermore, complicated analytical methods may be required to ensure complete polymer removal from the process fluid. Lastly, when adding charged polymer flocculants during the clarification process, process parameters such as mixing speed (rpm) and process duration should be carefully determined.^64^, ^65^, ^66^

### 2.2 Monoclonal antibodies—Classical mAbs, BsAbs, and ADC

Monoclonal antibody based biotherapeutics including bispecific antibodies (BsAbs) and antibody‐drug conjugates (ADCs) comprise a large proportion of the biopharmaceutical market.

In order to design optimal methods for the clarification of mAb‐based products, it is important to understand certain aspects of the cell culture fluid to be harvested. Several important factors that need to be considered in a harvest clarification process are described. First of all, upstream cell culture conditions are needed to consider, such as cell density, viability, pH, and osmolality for harvest stability. This stability causes stability of mAb products. Second, product characteristics should be considered, like pI value and product concentration (titer); finally, impurity profiles are important, such as HCPs, HCDs (Host Cell DNAs) and aggregates (compositions, turbidity).

Mi Jin et al.^67^ evaluated HCP impurity profiles in Chinese Hamster Ovary (CHO) cell culture by means of two‐dimensional difference gel electrophoresis (2D‐DIGE). According to the study, HCPs were found to be acidic (< pI 5–6) and < 75 kDa in molecular weight. Since the HCPs show a negative charge at a neutral pH range under normal cell culture conditions, positively charged depth filters can effectively remove them to an extent in the clarification process. As cell density increases in upstream processes, recent developments in harvest clarification strategy have included a two‐step depth filtration. Primary clarification is directed to the bulk removal of whole cells and solid particulates with coarser grade depth filters, while secondary depth filters remove finer impurities such as colloids as well as soluble HCPs and HCDs. In some cases, additional filtrations with microporous membrane filters are performed in order to reduce the burden on intermediate sterile filters and chromatography resins.

## 3 IMPORTANCE OF CLARIFICATION

From a quality perspective, clarification is considered to be the most variable unit operation at the upstream stage in the biopharmaceutical production process.^68^ For single‐use depth filters, guidance documents from the BioPhorum Operations Group (BPOG) describe typical concerns associated with the implementation of single‐use components in the biopharmaceutical manufacturing process.^68^ According to the guide, extractables and leachables (E&Ls) from the filters can present a negative impact on quality and/or process performance. In addition, the closer the process step is to the final drug substances (DS) or final drug products (DP), the higher the risks are rated on E&Ls from the filter components.

Since depth filters used in clarification are typically located upstream in the production process, extractables and leachables (E&L) from the depth filters used in this process are typically cleared in subsequent downstream process steps. A robust downstream purification process may minimize concerns associated with E&Ls from a quality perspective. Recently, cell culture processes have shifted toward higher cell densities to increase the yield of target products. As a result, clarification filters now include not only material of construction CE and DE filters, but also silica filter aids. In clarification steps that occur further downstream in the purification process, (E&Ls) may pose a significant risk.^69^ Consequently, the removal of these E&Ls adds additional pressure on downstream processes, including sterile filtration, chromatography, tangential flow filtration (TFF), and virus filtration. For this reason, the importance of risk assessments for clarification should be emphasized, and it is recommended that a QbD‐based approach be pursued.

Nitrosamines are chemical compounds and are described as probable human carcinogens based on animal studies. In mid‐2018, the EMA first became aware of the presence of nitrosamine impurities like NDMA (N‐nitroso‐dimethylamine) and NDEA (N‐nitroso‐diethylamine) in “sartans,” drug products used to regulate blood pressure, leading to market recalls. The root cause assessment indicated the cause to be the reaction of impurities like diethylamine and dimethyl amine with the nitrosating agent during the synthesis of the drug substance forming nitrosamine. Further cases came to light indicating the risk of nitrosamine formation in drug products during manufacture or storage as high in the presence of vulnerable amines (secondary or tertiary amines) and nitrosating agents in suitable conditions. The EMA released guidance in 2019 and called for a review of medicinal products manufactured through chemical synthesis by a 3‐step approach including (a) risk valuation, (b) confirmatory test, and (c) implementation of risk mitigation. The other regulatory authorities such as US‐FDA, ANVISA, and Health Canada followed a similar approach. In the last 5 years, the information related to nitrosamine contamination and its mitigation strategies has been continuing to grow. It was clear that a reaction between a vulnerable amine and a nitrosating agent would lead to nitrosamine formation and the risk factors are related to the manufacture of the active substance, finished product, and GMP‐related factors. The US‐FDA and EMA have indicated in their respective guidelines that nitrites are possible risk factors for nitrosamine formation during drug product manufacturing and storage in the presence of vulnerable amines in suitable conditions. Thus, drug product manufacturers are expected to closely work with their drug substance and excipient suppliers to perform a risk assessment of nitrosamine contamination in their finished products and establish appropriate mitigation steps or controls to address the same. Clarification filters are high in the upstream process of mAbs and any impurities coming from the depth filters are removed in the subsequent downstream unit operations such as chromatography and tangential flow filtration.^70^, ^71^, ^72^, ^73^

BPOG^68^ presents the concept of leaching propensity in drug production, emphasizing an increasing risk as the process progresses from upstream activities to the final drug product. This progression is referred to as the “Distance along Production Stream. (DAS)” Key factors that contribute to this elevated risk include exposure temperature, exposure duration, process fluid interaction, and the dilution ratio, which considers the surface area relative to volume. In the upstream phase, activities such as working with cell banks, vial thawing, inoculum preparation, expansion, production, harvest, and plasma processing take place. As the process moves into the purification phase, techniques such as affinity chromatography, viral inactivation, ion exchange chromatography, viral filtration, and ultrafiltration or diafiltration are utilized. Following purification, the focus shifts to the bulk drug substance phase, which involves final filtration, sterile filtration, and the storage of the bulk drug product. The final stage consists of formulation, filling, and finishing, which includes bulk drug product storage, potency adjustment, sterile filtration, filling, and lyophilization. As the drug moves through these stages, the risk of leaching increases, peaking in the final drug product.^68^

We have summarized Table 2, ^68^ presents an example of a leachables risk assessment (RA) model that can be applied to the clarification process. Traditionally, many pharmaceutical companies have considered the harvest step to be of low risk. However, this perception may shift if the DAS and process fluid interaction (PFI) are higher than previously understood for clarification.

**TABLE 2:** Example leachables risk assessment model by BPOG.^68^

Consideration	Ratings	Level description	Weight
DAS	1	Upstream: For example, working cell bank, vial thaw, inoculum, expansion, production, harvest, plasma and solution preparation	0.40
	3	Purification: For example, filtration, chromatography, viral inactivation, viral filtration and UF/DF	
	5	Bulk drug substance: For example, formulation, 0.22 μm filtration, BDS storage	
	9	Final formulation, fill/finish: For example, bulk drug product storage, potency adjustment, sterile filtration and filling	
Exposure temperature (ET)	1	<0°C	0.15
	3	0 to 8°C	
	5	>8 to 30°C	
	9	>30°C	
Exposure duration (ED)	1	Transient (≤60 min)	0.15
	3	Short (≤24 h)	
	5	Medium (≤7 days)	
	9	Long (>1 week)	
Process fluid interaction (PFI)	1	Limited penetration into polymeric component (i.e., water)	0.15
	3	Low solvation power or low penetration of polymeric component (e.g., neutral pH without organics, surfactants, etc.)	
	5	Medium solvation power or medium penetration of polymeric component (e.g. surfactant, low‐concentration organics, high/low pH solutions without organics/detergents)	
	9	High solvation power or high penetration of polymeric component	
Dilution ratio (DR)	1	<1 × 10–^03^ m^2^/L	0.15
	3	1 × 10^−02^ to 1 × 10^−03^ m^2^/L	
	5	1 × 10^−01^ to 1 × 10^−02^ m^2^/L	
	9	>1 × 10^−01^ m^2^/L	
Leachables risk rating (LRR) calculation	Calculated by the following equation: LPR = DAS*Weight (0.4) + ET*weight (0.15) + ED*weight (0.15) + PFI*weight (0.15) + DR*weight (0.15) Possible range: 1.0 to 9.0		
Leachables risk rating levels	**6.3 to 9.0: High** **3.7 to 6.2: Medium** **1.0 to 3.6: Low**		

Table 3 illustrates how results can change when applying the leachables RA model to clarification.

**TABLE 3:** Change of leachables RA model.

RA of clarification process for present						RA of clarification process for future expect				
	Rating	Level description	Weight	Score			Rating	Level description	Weight	Score
DAS	1	Upstream	0.4	0.4		DAS	3	Purification	0.4	1.2
ET	5	>8 to 30°C	0.15	0.75		ET	5	>8 to 30°C	0.15	0.75
ED	3	Short (≤24 h)	0.15	0.45	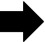	ED	3	Short (≤24 h)	0.15	0.45
PFI	1	Limited penetration into polymeric component (i.e., water)	0.15	0.15		PFI	3 or 5	Low solvation power or low penetration of polymeric component	0.15	0.45 or 0.75
								Medium solvation power or medium penetration of polymeric component		
DR	9	>1 × 10–01 m^2^/L	0.15	1.35		DR	9	>1 × 10–01 m^2^/L	0.15	1.35
**Total**				**3.1 low**		**Total**				**4.5 medium**

The distance along production stream (DAS) of the clarification process shifts from upstream to purification because a depth filter is used for clarification. Specifically, for the filtration stage in purification, the rating is set at 3, and with an adjusted weight of 0.40, this results in a final score of 1.2. Additionally, the PFI may change due to the composition of the buffer used in the clarification process. The buffer could be composed of low or high pH or conductivity, which could increase the PFI score to 3–5. Applying this with a weight of 0.15, the resulting score would be either 0.45 or 0.75.

## 4 HOW TO APPLY A QbD CONCEPT TO THE CLARIFICATION PROCESS

### 4.1 Roadmap

The FDA and EMA emphasize the importance of QbD (Quality by Design) in ensuring consistent product quality in biomanufacturing. QbD is a systematic approach that combines scientific principles and risk assessment to optimize manufacturing processes, providing guidelines that manufacturers must follow.^74^, ^75^, ^76^, ^77^, ^78^ The concept of QbD involves defining a Quality Target Product Profile (QTPP) to identify Critical Quality Attributes (CQAs) that need to be controlled within specific limits. Risk assessments are then used to identify potential risks and determine Critical Process Parameters (CPPs) and CQAs that impact product quality.^79^ Design of Experiments (DoE) techniques are used to systematically study and optimize process parameters, helping to improve the relationship between these parameters and product quality. By implementing QbD principles, biomanufacturers can enhance product quality, optimize processes, and ensure regulatory compliance throughout the product lifecycle.^79^, ^80^, ^81^

### 4.2 QTPP, CQA, CPPs, design space, and control strategy for clarification

**FIGURE 3:**
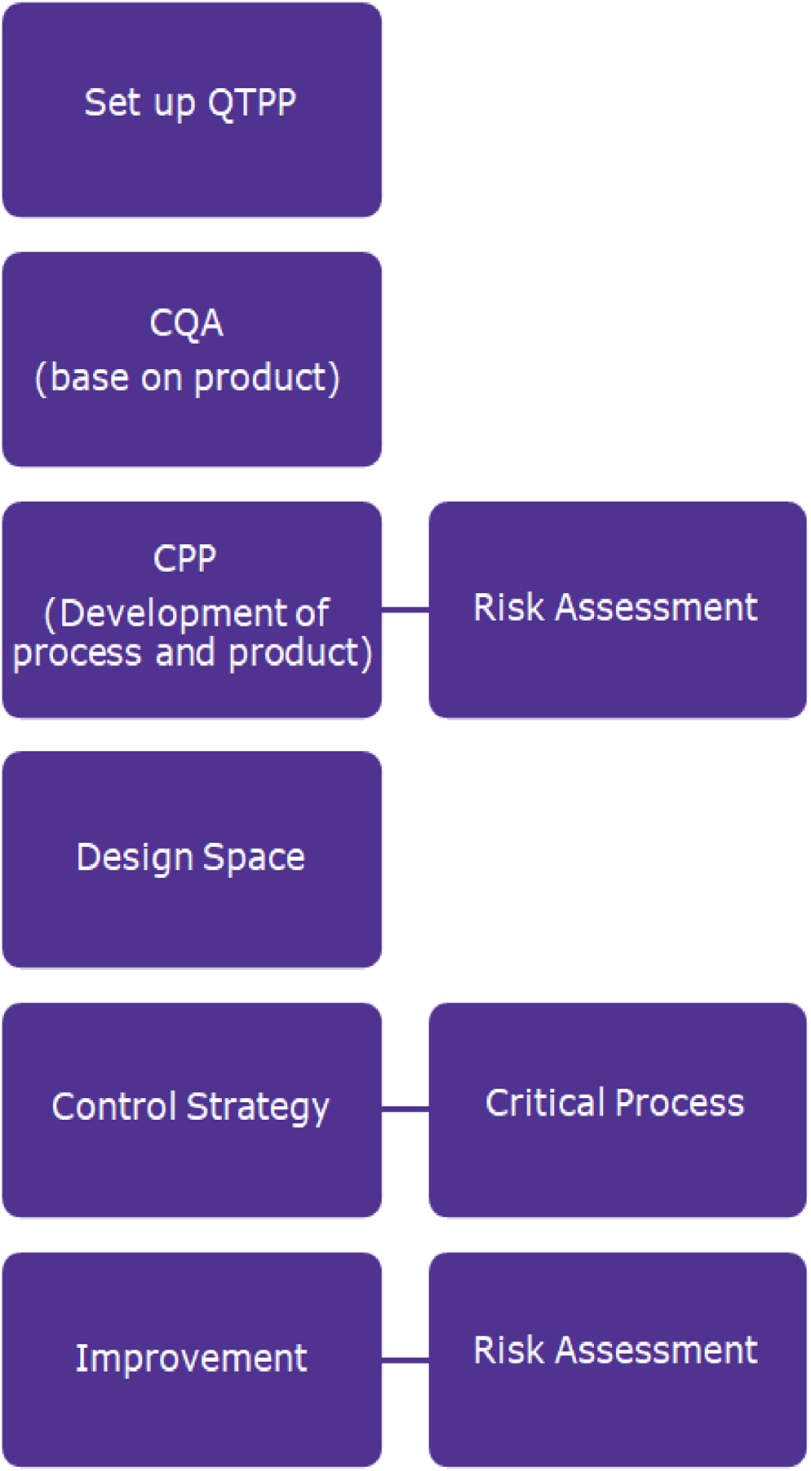
Elements of QbD.

QbD plays a crucial role in biomanufacturing, especially in the clarification step, by removing impurities and improving product quality in a cost‐efficient way. By leveraging risk assessments, control strategies, and techniques like Design of Experiments (DoE), manufacturers can reduce process variability and enhance consistency. Defining the Quality Target Product Profile (QTPP) and Critical Quality Attributes (CQAs) ensures safety and efficacy, while control strategies and design space further support quality throughout the product lifecycle.^82^, ^83^ Figure 3 shows elements of QbD.

#### 4.2.1 QTPP (quality target product profile)

The QTPP guides the desired qualities of dose, concentration, and impurities. The QTPP also sets acceptable limits for impurities, and effective clarification processes will minimize those impurity levels. By aligning the clarification process with the QTPP, manufacturers may optimize processing parameters to achieve the desired attributes, ensuring product quality and therapeutic efficacy.^84^, ^85^

#### 4.2.2 CQA (critical quality attribute)

Clarification processes should consider that the potency, purity, and content of the drug substance are critical factors in ensuring the quality of the final biopharmaceutical product. An effective clarification process should not impact the drug potency but may remove impurities that could potentially interfere with the therapeutic effect of the drug substance.^86^, ^87^ Typical clarification processes employ techniques such as centrifugation, depth filtration, or a combination of both to eliminate impurities. By considering the potency, purity, and product recovery of the clarification step, manufacturers are able to implement appropriate control strategies; perform risk assessments to consistently achieve the desired quality attributes.^88^ This ultimately ensures the production of high‐quality biopharmaceuticals that deliver the intended therapeutic effect, comply with regulatory requirements, and prioritize patient safety.^89^

#### 4.2.3 CPP (critical process parameter)

CPPs in the clarification process include factors such as centrifugation speed, filtration parameters (such as flux), buffer conditions (pH, osmolarity, etc.), inlet pressure, delta pressure across the filter, and temperature control. These parameters have a significant impact on the effectiveness of solids removal, impurity clearance, product recovery, and subsequent product stability.^90^, ^91^ Determining the CPPs specific to the clarification process is based on process knowledge, risk assessment, and a scientific understanding to ensure consistent product quality.

#### 4.2.4 Risk assessment for the clarification process

Risk assessment is a crucial component of the QbD approach. It involves identifying, analyzing, and managing potential risks that may arise during product development and manufacturing. This process is particularly important in complex processes, such as biopharmaceutical manufacturing, where critical decisions must be made at every stage of product development to maintain consistent product quality, ensure patient safety, and minimize business risk. Failure modes and effects analysis (FMEA), hazard and operability study (HAZOP), and fault tree analysis (FTA) are three main tools frequently used for risk management. FMEA evaluates potential defect modes and their effects on a product or process to improve system reliability.^92^ HAZOP systematically reviews process designs to identify operational and safety issues, specifically using “guide words” to identify variations in a process. It tracks the causes of potential defects or errors within a complex system and represents them in a visual tree structure to evaluate the reliability of the entire system. FMEA, along with other tools, can help to ensure the quality and safety of the product.

FMEA is a tool used to identify and prioritize potential defects in the early stages of product or process design.^93^ It is important to note that FMEA is not the only tool available for this purpose. This approach enables resources to concentrate on the most crucial issues by systematically analyzing the impact of each failure mode on key functions and allocating the number of risk priorities based on the likelihood and severity of occurrences. FMEA also emphasizes problem prevention, which reduces modification costs and enhances the safety and reliability of products. This approach to prevention can improve quality control during product development, leading to cost savings and increased customer satisfaction.

#### 4.2.5 Fishbone tree for clarification

**FIGURE 4:**
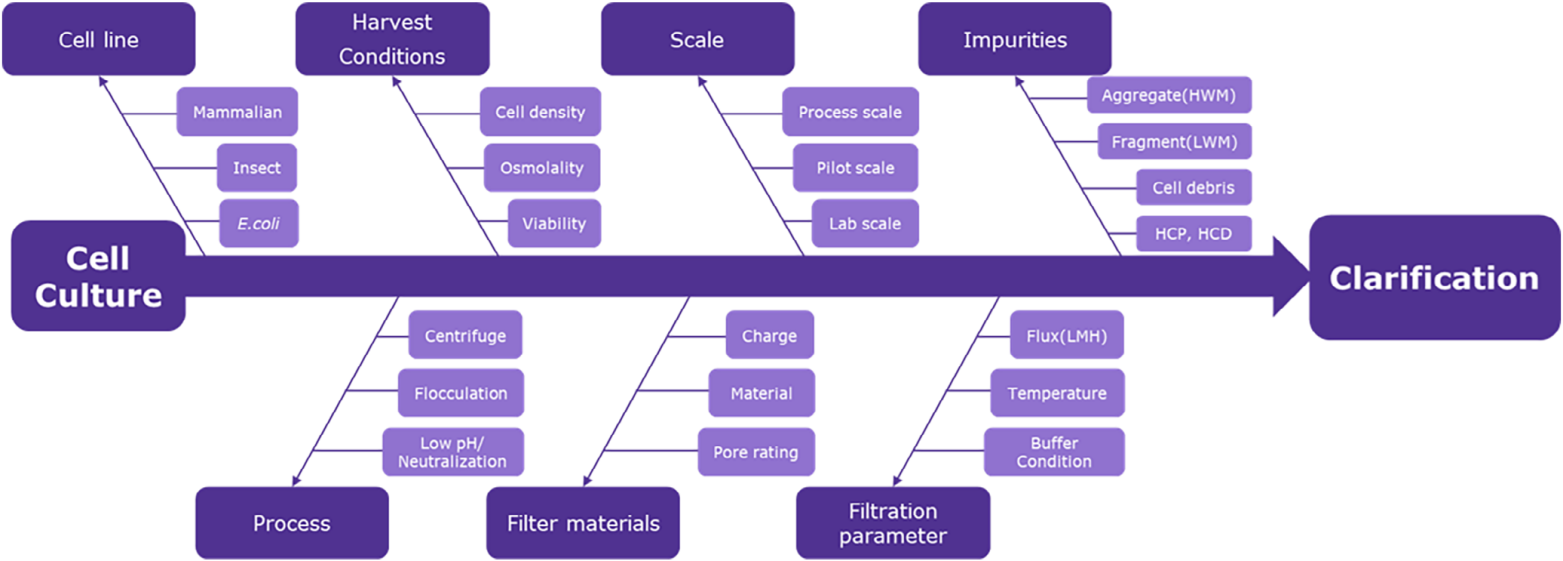
Example of fish‐bone tree for clarification process.

The Fishbone diagram (Figure 4), also known as an Ishikawa diagram, can be used to identify factors that affect the clarification process. This tool visualizes cause‐and‐effect relationships and is particularly useful in QbD for identifying and classifying potential problems or causes of quality defects in a product or process.^76^ Fishbone Diagrams can be used to systematically decompose complex problems and identify key causes, increasing the effectiveness of quality control.^94^ This fragment of a fishbone analysis displays the usefulness of an approach that considers various factors during the development phase of the QbD's risk assessment (RA) and Control Strategy. The factors that affect clarification, such as cell line, process, filter materials, scale, and impurity, are crucial when optimizing a successful clarification process. The parameters are categorized into different parts of the cell line, cell culture information, scale, impurities, process, filter materials, and filtration parameters, as shown in Figure 4.

#### 4.2.6 FMEA for risk assessment

**FIGURE 5:**
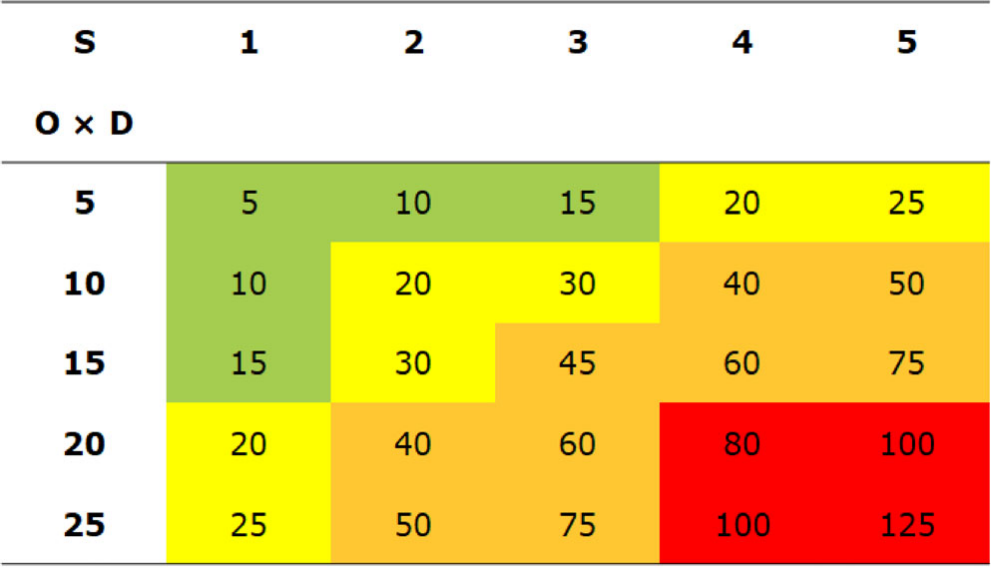
Example of risk matrix determination based on RPN.

To assess and prioritize the risks associated with process robustness and productivity, a thorough analysis was conducted that combined historical data with a deep understanding of the process. This analysis utilizes an FMEA methodology.^95^ Within this framework, each process parameter was scrutinized for its probability of failure, the severity of its potential impact, and the likelihood of its detection. These aspects were evaluated on an individual basis and then combined through multiplication to yield a Risk Priority Number (RPN) for each parameter, effectively quantifying the associated risk level.^96^ The total RPN was calculated using the formula: RPN = S × O × D. Figure 5 describes an example of risk matrix determination based on RPN. As illustrated in Figure 5, the RPN varied between 1 and 125. Furthermore, the average RPN for each potential Failure Mode (FM) category was determined.^97^, ^98^ Table 4 shows an example of results of the FMEA for clarification‐relevant CQAs. Each CQA was assessed on a scale from 1 to 5 for probability, severity, and detectability. A score of 1 indicates no potential risk, a score of 3 denotes a moderate or controllable risk, and a score of 5 signifies a considerable risk.

**TABLE 4:** Example of results of the FMEA for clarification‐relevant CQAs. Each CQA was assessed on a scale from 1 to 5 for probability, severity, and detectability.

Possible CQA	Probability	Severity	Detection	RPN
Low yield	2	5	4	40
Filter plugging	3	2	2	18
Endotoxin level	3	3	4	36
HCP level	3	3	4	36
HCD level	3	3	4	36
…	…	…	…	…

*Note*: A score of 1 indicates no potential risk, a score of 2 indicates low moderate risk, a score of 3 denotes a moderate or controllable risk, a score of 4 indicates high moderate, and a score of 5 signifies a considerable risk.

## 5 PROCESS DEVELOPMENT FOR CLARIFICATION

### 5.1 Considerations for early process development

Many depth filters commonly used for clarification applications in bioprocessing comprise cellulose or polypropylene fibers as a matrix. These materials provide a complex network structure and a high surface area to the filter material. Along with the fibrous filter media, appropriate filter aids, such as diatomaceous earth, activated carbon, silica gel, or perlite, are embedded within the media to provide increased adsorption of colloidal material and soluble impurities.^99^, ^100^ The fibers and the filter aid are bound by a polymeric resin binder, which also confers a positive surface charge to the filter media. Depth filtration media perform via a combination of size exclusion mechanism and charged interaction, either by sieving on the surface of the depth filter or within the porous matrix of the depth filtration media. In addition, positively charged depth filter media are also effective at binding negatively charged impurities such as HCP^101^/DNA,^102^ viruses,^103^ and endotoxins^104^ by an electrostatic adsorption mechanism. The filter aid and resin binder type may impart a range of mechanical and electrostatic removal mechanisms during the clarification step, including hydrophobic adsorption, hydrogen bonding, and mixed‐mode binding interactions.

The selection of an appropriate depth filtration media is an important consideration during the initial process development for the clarification step. The pore size and materials of construction of the selected filter can affect the process capacity, product yield, and soluble impurity profile. Manufacturers typically offer a range of depth filtration media grades spanning a range of nominal pore sizes. For a given biological feed stream, the smallest particles (<1 μm) include negatively charged cell debris and colloidal material that can be removed by a combination of size exclusion and adsorption with the positively charged depth filters. The nominal pore size rating of the filter should be carefully selected. If the nominal pore size rating of the selected depth filter is too large, small particles may not be retained, resulting in the rapid clogging of downstream bioburden reduction filters. Conversely, if the nominal pore size rating of the selected depth filter is too small, there may be rapid fouling of the depth filter since large particles will deposit on the depth filter surface as a cake, and this may greatly increase fluid resistance.^105^ The selection of the appropriate nominal micron rating for the depth filtration step is often determined empirically with consideration of the cell culture harvest density, % viability, and the impurities that are to be removed. If the targeted impurities are cells and cell debris, a good starting point is a depth filter with a relatively large nominal pore size, whereas secondary clarification or haze removal may require a depth filter media with a strong positive charge and smaller nominal pore size rating. A typical clarification process for mAb production may employ a two‐stage clarification process including a primary clarification to remove whole cells and cell debris, followed by a secondary clarification process for turbidity reduction and to remove low levels of soluble impurities.^106^

### 5.2 Establishing CPPs and CQAs

CPPs are parameters that affect CQAs. The main goal of the post‐harvest clarification process is to remove large particles such as cells and cell debris, thereby reducing the burden on downstream processes.^107^

In a standard clarification process utilizing depth filters, both the product yield and purity are of utmost importance. Influencing factors include harvest conditions such as Total Cell Density (TCD, cells/mL), viability (%), and harvest duration.^107^ Other relevant parameters are flux (Liter per square meter per hour, LMH)^90^, ^91^, ^106^ osmolarity (mOsm/kg)/conductivity (mS/cm),^101^ temperature (°C), and inlet pressure (psi).^108^, ^109^ Certain physical process parameters may be regulated by the buffer conditions. Inlet pressure, typically operated at a fixed/controlled level, is critical as it directly influences shear stress. Cell shear stress can vary with an increase in flow rate and fluid viscosity. As the viscosity of the fluid increases and the flow rate becomes faster, the resistance from the filter also increases, which can lead to an increase in inlet pressure and cell lysis, which increases the impurity of the clarified filtrate. It is suggested to control the inlet pressure as a potential critical process parameter (CPP) in the clarification process.^110^ Excessive shear stress can alter cell structure, leading to higher impurity levels and reduced product purity. These parameters can be designated as CPPs for the clarification process. Product yield is determined by comparing the amount of the target product before and after the clarification process. Product titer and % recovery are critical evaluation metrics. It is also essential to verify that purity meets specified levels for impurities such as HCP, HCD, aggregates, and fragments. The specified levels for impurities after the clarification step would be dependent on the capabilities of the subsequent downstream processing steps to remove them to satisfactory levels in the drug substance. The requirements of the clarification step may vary as a result. It may also be worthwhile to note that the degree of clearance of impurities may also directly impact the performance of subsequent purification steps, such as resin lifetime of the chromatography capture step. When the cell culture harvest condition is high cell density but low viability, HCP/HCD levels are higher in comparison to low cell density and/or higher viability cell culture. Additionally, the filtrate turbidity, which is linked to host cell protein contamination levels,^101^ is considered a risk factor for downstream processing as it can lead to clogging in sterile filters.^101^, ^111^ These factors are established as potential CQAs, which may vary depending on the host cell. For instance, when using *E. coli* as the host, endotoxin levels are a critical CQA.

The levels of HCP and HCD can be established as purity criteria, which are influenced by the degree of cell lysis. Lower cell viability results in higher concentrations of these substances in the cell culture fluid, complicating their removal in downstream processes.^101^ HCP and HCD, primarily produced during cell culture, impact the safety and efficacy of drugs. Consequently, regulatory agencies set limits and regulate the content of HCP and HCD to prevent infections, allergic reactions, and reduce the risk of diminished drug effectiveness or the occurrence of side effects. These substances can be at least partially removed through depth filtration.

It has been reported that the removal efficiency of impurities in the depth filtration process can depend on flux.^90^, ^91^, ^106^ When impurities are removed through an adsorption mechanism, a longer residence time on the filter can aid in their removal, leading to improved filtrate quality. However, the effect of flux is diminished if the impurities are removed solely by a sieving mechanism, where the impurities accumulate on the filter surface (cake formation) or block the pore size (Complete pore‐blocking).^106^ Flux is considered to be a critical parameter in the depth filtration process, especially when using adsorption mechanisms such as depth filters containing activated carbon. Determining the optimal flux is essential for effectively removing impurities by these methods.^101^

Loss of the target product during depth filtration can be attributed to two factors. If the product has a negative charge, it may bind to the positively charged filter by an electrostatic interaction. To prevent adsorption losses, it is important to check the product's isoelectric point (pI) and the buffer's pH. Maximizing recovery from the clarification step is crucial, and product yields may be improved through the use of air blowing or buffer chasing to recover any entrapped process fluids from the depth filtration devices. It is noted that while air blowing may not recover all the product due to the filter's hold‐up volume, chasing the filters with a suitable buffer solution can significantly improve product yield. A proper salt concentration should be maintained during the recovery flush to prevent additional yield losses.^109^

Table 5 shows a risk assessment for establishing CPPs and CQAs of the clarification process by referring to a cause and effect matrix. The cause and effect matrix also includes an importance rating for evaluating the effects and correlation scores between causes and effects. A high importance rating indicates if the cause has a strong correlation to the described effect.

**TABLE 5:** Expected cause and effect matrix for clarification process.

			Effect (possible CQAs)								
Rating of importance to clarification		9	9	1	3	7	2	9	9	9	Calculation
Cause		HCP	HCD	Aggregate (HWM)	Fragment (LWM)	Cell, cell debris	Product concentration	Recovery	Turbidity	Filter loading capacity (L/m^2^)	Total value
(CPPs)											
Harvest conditions	Cell density	R	R			R	R		R	R	45
	Particle size	R	R			R			R	R	43
	pH	R	R		R		R	R			32
	Osmolality (mOsm/kg)	R	R				R				20
	Harvest time	R	R			R	R				27
Physical parameter for clarification process	Flux (LMH)	R	R						R	R	36
	Conductivity (mS/mL)				R			R			12
	pH				R			R			12
	Osmolality (mOsm/kg)	R	R			R	R				27
	Temperature			R							1
	Inlet pressure	R	R			R		R	R	R	52

*Note*: Calculation total value for each Cause = Sum the rating of importance from related effect. Example: Cause 1 (cell density) value = Related rating of importance (HCP + HCD + Cell/Cell debris + Product concentration + Turbidity + Filter Loading capacity [L/m^2^]) = 9 + 9 + 7 + 2 + 9 + 9 = 45. High importance value: Red (41–58). Medium importance value: Yellow (23–40). Low importance value: Green (0–22).

Abbreviation: R, related.

Table 5 describes a cause‐and‐effect relationship between CPPs and CQAs. Conductivity is a representative parameter indicating the concentration of ions, while osmolarity primarily refers to the concentration of solutes that help maintain osmotic pressure in cells. Therefore, the contribution to the osmolarity can include not only ions but also glucose, amino acids, and other substances. If osmolarity changes during the clarification process, it can lead to cell lysis, which in turn can affect the quality of the filtrate. One of the mechanisms of charged impurity removal is based on ionic interaction with a positive charge resin binder, which is a component of depth filter; hence, conductivity can influence the impurity clearance.

### 5.3 Process development by DoE to define a design space for manufacturing

Sections 5.1 and 5.2 describe the CPPs and the potential for CQAs to be influenced by impurities during the clarification process. These insights contribute significantly to the establishment of a design space for the clarification process. Traditionally, in the development of biotherapeutics, clarification has been regarded as an integral part of the Upstream Harvest process. Initial focus was on the collection of cell culture, with research directed toward optimizing conditions for continuous centrifugation. Subsequent purification processes employed depth filters or bioburden reduction filters, emphasizing filter application studies over an in‐depth examination of process parameters. With recent advancements in process development technology, the crucial factors within the clarification process, as identified in Sections 5.1 and 5.2, can be pinpointed. Moreover, an improved understanding of the effect of process impurities on CQAs has been acknowledged. This recognition opens the possibility of defining a design space for the clarification process. Table 6 describes the steps involved in the QbD principle for product development. For the process development, DoE and the One Factor At a time (OFAT) method could be used for screening of process parameters and materials.

**TABLE 6:** Three steps involved in QbD principle for product development.

(1) Development of new molecular entity	(2) Manufacturing	(3) Control strategy
Preclinical study Nonclinical study Clinical study Scale‐up Submission for Market Approval	Design space Process analytical technology Real time quality control	Risk based decision Continuous Improvement

Design space, as outlined in ICH Q8 (*R* ^2^),^112^ encompasses the multidimensional interplay and interaction of input variables (e.g., material attributes) and process parameters proven to ensure quality. Operating within this design space is not deemed a change, and thus does not necessitate post‐approval modifications. Conversely, deviating from the design space is viewed as a change, typically triggering a regulatory process for post‐approval amendments. The proposal of a design space is the responsibility of the applicant and must undergo regulatory evaluation and approval.

The design space is not a checkbox requirement for the successful implementation of QbD; the product and process knowledge can be successfully obtained and implemented even if there is no formal establishment of design space.

The design space defines the operational boundaries of process parameters and their impact on CQAs across manufacturing stages. It includes the acceptable ranges for all CPPs and associated CQAs, established through multivariate analysis and experimental designs. The scope of the design space can be limited if multiple CQAs are affected by the same CPPs. It is crucial for product conceptualization and aligning with the QTPP. Prior knowledge, preliminary risk assessment, and various models help identify key variables for the design space, ensuring consistent product quality. Univariate data inform these acceptable ranges; for existing products, multivariate models assess historical data to refine the design space.^89^ In order to increase the robustness of the manufacturing process and minimize the number of rejected batches, while respecting the quality requirements, the QbD approach was introduced. QbD is based on process understanding and typically relies on applying a DoE framework in order to experimentally cover a broad operating space during the design stage. The comprehensive experimental investigation provides the subset of the operating space, called the design space, within which the product quality requirements are met.^113^

Therefore, by consolidating the situations considered in Process Development as identified in Sections 5.1 and 5.2 into CPPs and CQAs or QTPP, they align as shown in Table 5. Through this, it is possible to construct the Design Space using the DoE or OFAT method, and systematic and efficient process development is anticipated through DoE.

### 5.4 Example of a study for the design space for clarification

The study for the design space could be conducted in two steps. The first step, which occurs during the early stages of development, involves screening filters or parameters. The second step, during the later stages of process development, focuses on process characterization. In the first step, one could utilize OFAT or Screening DoE primarily for selecting filters and flocculation methods (including primary and secondary depth filters). In the second step, one could employ a response surface methodology (RSM) of DoE to verify the parameters.

#### 5.4.1 Process development in early stage—Harvest condition optimization and depth filter screening study

##### Harvest condition optimization study

At the early stage of process development, which ranges from discovery to pre‐clinical phases, there are numerous options available for optimal process design. During this stage, researchers involved in process development often consult academic articles, studies, and suppliers of commercialized depth filters for guidance. As described in Table 1 and Section 5.1, which outline the types of filters and their significance, the initial step in process development is the screening study for filters. Before initiating the filter screening study, it is crucial to optimize the Upstream Process (USP), particularly the harvest conditions, including VCD (cells/mL), viability (%), pH, conductivity, osmolality, and more. These harvest conditions significantly influence the clarification process, as mentioned in Sections 5.1 and 5.2. In filter screening studies, researchers typically focus solely on filter throughput (L/m^2^), filter retention (filtrate turbidity), and product concentration (yield %). However, if a researcher is incorporating the QbD concept into the clarification process, there are several additional focal points to consider, such as possible CQAs, including HCD, HCP, endotoxins, aggregates, and more. Thus, at the early stage of process development, it is important to consider representative CQAs for clarification, including product concentration (yield %), HCP levels, and endotoxin content.

Most biopharmaceutical companies prioritize achieving higher product concentration and product yield, % in the USP during process development. To optimize the design space for USP, studies are needed on variables such as pH, dissolved oxygen (DO %), glucose concentration (g/L) and lactate levels, culture temperature (°C), and timing of temperature shifts. According to DoE principles, these parameters could serve as X factors while the product concentration (yield, %) could be the Y response. Consequently, many biopharmaceutical companies conduct DoE studies.^114^

As described in this paper, the parameters focus solely on the culture conditions. However, most companies may not want to emphasize the harvest conditions prior to the clarification step, which could include critical factors such as VCD (cells/mL) and viability (%) at the time of harvest, levels of lactate, pH, conductivity, and osmolality. These parameters can significantly impact product quality. It is important to note that the quality concerns are not limited to product concentration (yield, %); they also include impurities such as HCP/HCD, endotoxins, and aggregates.

Bracewell has published case studies regarding the harvest time parameters that affect host cell protein clearance.^115^ The results showed that as the cultivation period extended, the levels of post‐protein A HCP impurity increased, indicating a negative correlation between product quantity and purity. Specifically, the amount of product increased by 28% from Day 14 to Day 17, while HCP levels increased by 75% during this period.

Bracewell had also performed a DoE to explore the impact of culture conditions on the trade‐offs between titer and product quality. His study revealed that conditions promoting antibody production also resulted in higher levels of post‐protein A HCP. Based on this data, a contour plot was generated to illustrate the pH and temperature ranges that provide a suitable process window, balancing high titer and low HCP levels upstream and downstream.^115^

Therefore, a comprehensive study of the harvest conditions is necessary; it should be completed before optimizing the clarification process development.

For the study of harvest conditions, having team members with substantial experience and knowledge in process development can significantly shorten the duration of the study. Experienced personnel, for instance, might not require extensive preliminary screening using designs such as full factorial DoE. Instead, they can leverage their deep understanding of the process to predict outcomes and directly employ more specialized experimental designs, such as Response Surface Methodology (RSM) or custom‐designed DoE. This streamlined approach not only expedites the research but also enhances the efficiency of experimentation by utilizing the expertise available within the team.

However, if the product is a “new drug product” or if the company lacks experience or knowledge in process development, numerous studies will be necessary. A full factorial design can be implemented for both screening parameter studies and process characterization studies. Consequently, it may take more time than other products before this product can be released.

##### Filter screening study

Table 7. describes an example of a filter screening study.

**TABLE 7:** Example of filter screening study.^116^

Categorical	Filter combination for clarification process			Possible CQAs		
	1	2	3			
Primary clarification	D0HC	D0SP	CS20MS	Product concentration (yield, %)	HCP/HCD	Aggregates
Secondary clarification	X0HC	X0SP	A1HC			

The goal of primary clarification filters is a bulk removal of whole cells, cell fragments, and large particles. Primary depth filters present a pore size that is larger than secondary depth filters. The purpose of secondary depth filters is the removal of small particles (<1 μm), and colloidal protein agglomerates. After the filter screening study, a filter sizing study may be conducted on a lab scale. This study helps to determine the filter configuration and provides information on the process flux and the ratio between primary and secondary clarification filters. Based on this information, a parameter screening study can then be conducted.

#### 5.4.2 Process development after early stage—Clarification parameter screening and process characterization study

After completing the filter screening study, pharmaceutical companies may initiate a parameter study, particularly during the pre‐clinical phase as they prepare for clinical phase studies. It is important to note that the product used in the clinical phase is nearly identical to commercial products. Therefore, the development of manufacturing parameters is conducted promptly to ensure no delay in process optimization. The parameter screening is carried out using the DoE method. As a core principle of DoE, parameter screening serves as an experimental approach to effectively identify and optimize variables that significantly impact complex processes or systems. This method is particularly valuable for swiftly assessing the effects of multiple input variables on process outcomes and is crucial for understanding the significance of these variables in the early stages of development.

In addition, a process characterization study for defining a design space involves a series of experimental design and analysis techniques. These techniques help define the impact of each parameter and its acceptable ranges, focusing on identifying conditions that maximize productivity and efficiency while ensuring product quality. The primary goal of establishing design space is to guarantee that the process consistently meets quality standards and provides operational flexibility, thus supporting the efficient progression from pre‐clinical to clinical development phases.^117^

The parameters that are classified as possible critical process parameters are detailed in Section 5.2. Studies on these parameters may be divided into two parts: the first part focusing on harvest conditions controlled during the USP and the second part on clarification conditions that could be classified during Downstream Processing (DSP).

##### Clarification condition

After defining a harvest condition that focuses on product yield and impurities, the upstream process fluid (harvested cell culture fluid, HCCF) may be representative for the clarification process. The filter combination and recommended process flux (LMH) can be determined by a filter screening study followed by a filter sizing study at the laboratory scale. As described in Section 5.2, Table 8 describes CPPs and CQAs by means of a cause and effect matrix. From this information, the researcher could have studies for the optimal process; then a design space study could be set up by DoE from derived results.

**TABLE 8:** DoE preparation for the design space of a clarification process.

Possible CPPs	Set point	Range	Possible CQAs
Flux (LMH)	60	40–120	Product concentration (yield, %), HCP/HCD level, endotoxin, aggregates
Osmolality (mOsm/kg) for buffer	300	0–600	
pH for buffer	7	7–8	
Conductivity (ms/cm) for buffer	10	0–20	

Sections 4, 5.1 and 5.2 describe the important considerations for a well‐defined clarification process, including the control CPPs and their effect on the CQAs. Among the possible CQAs, product concentration (yield, %), HCP/HCD, and Aggregates are the most important CQAs. The CPPs will include Process Flux (LMH) and buffer conditions (Osmolality, Conductivity, pH). Section 5.4.1 describes the initial process development for clarification, such as screening parameters and quality attributes. Based on all this RA and initial study, the study about the proven acceptable process parameter could be conducted. If there is limited information or knowledge of the clarification process, a parameter screening study (Full factorial Design, DoE) should be employed to fully evaluate the parameters that affect the CQAs. An RSM or Custom design DoE should then be used for setting the range of the parameters for the design space. Table 8 shows an example of a DoE study, with CPPs that include all parameters and CQAs.

It may be difficult to verify, satisfy, and control all CPPs and to evaluate their effect on the CQAs. In addition, while there is a significant amount of information available for classic mAbs for process development, there is much less information available for bi‐specific mAb, and Fc‐fusion products. For these situations, biopharmaceutical companies may need to obtain additional information from published articles or their experience from related molecules in order to help define the design space.

Tables 9 and 10 show another example for the clarification process DoEs (process parameters include process flux and osmolality).

**TABLE 9:** DoE for design space of clarification process.

Possible CPPs	Set point	Range	Possible CQAs
Flux (LMH)	80	40–120	Product concentration (yield, %), HCP
Osmolality (mOsm/kg) for buffer	300	0–600	

**TABLE 10:** RSM DoE (central composite design) randomized run order of example.

Run order	Pattern	Flux (LMH)	Osmolality (mOsm/kg)
1	00	80	300
2	00	80	300
3	−+	40	600
4	++	120	600
5	0a	80	0
6	+−	120	0
7	0A	80	600
8	00	80	300
9	A0	120	300
10	a0	40	300
11	−	40	0

**FIGURE 6:**
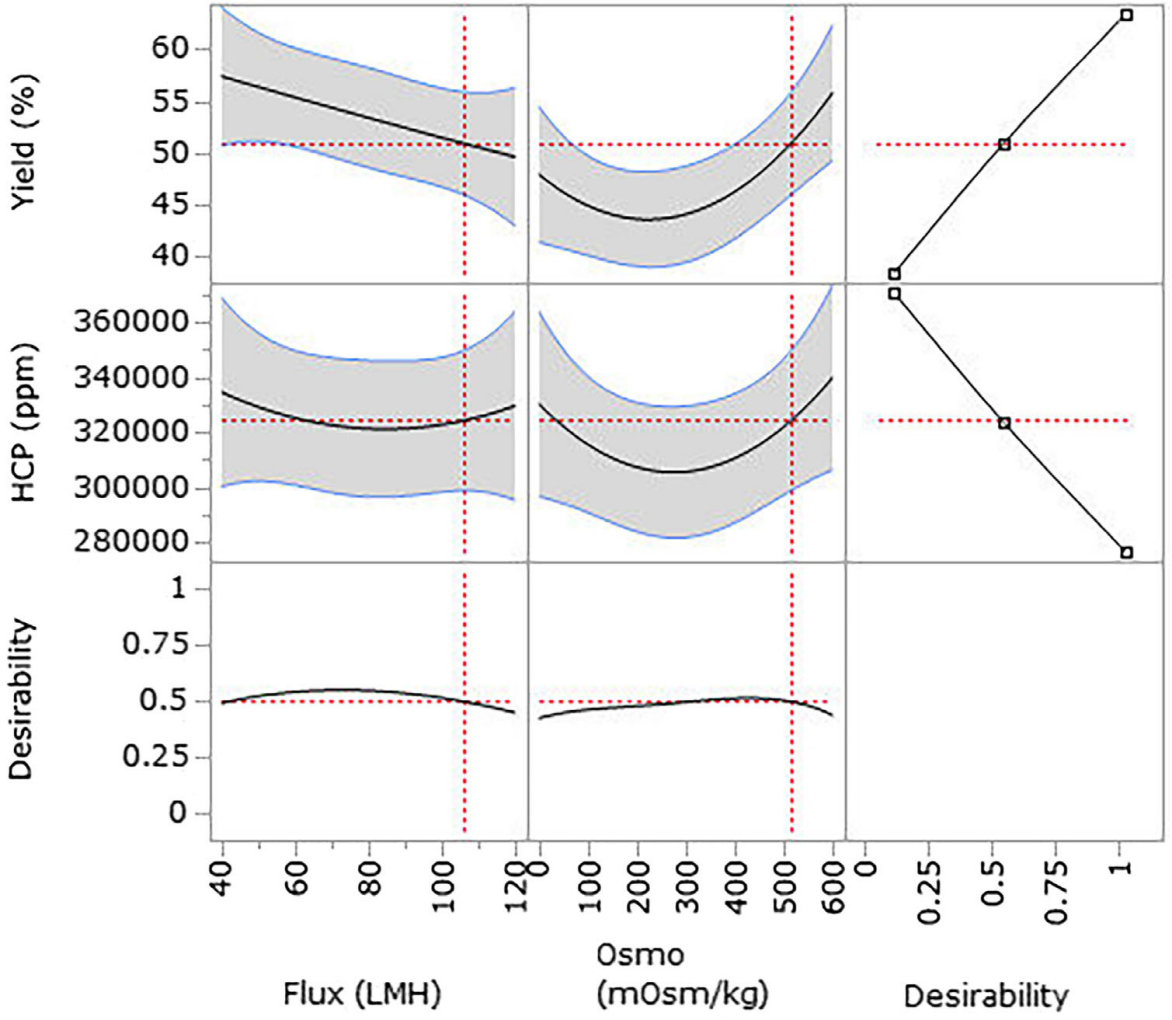
Example of prediction profiler for clarification.

Table 10 illustrates a compact design for process parameters. Based on the results from such a DoE, researchers can create a custom DoE using statistical software (JMP or Minitab). After conducting experiments in the specified run order, the results are analyzed using Analysis of variance (ANOVA), which allows the identification of a meaningful range of CPPs that affect the CQAs. Additionally, the verified range can be determined using a Prediction Profiler. The Prediction Profiler forecasts the range for the target based on desirability. Generally, if the desirability score is high, the likelihood of achieving optimal results is also higher. Figure 6, ^117^ displays the Prediction Profiler by JMP software. The JMP software is statistical program and is used for process development in many biopharmaceutical companies. JMP has basic statistic calculation such as ANOVA, *t*‐test, Equal variance test, etc. This program is also used for DoE to make design space for prediction profiler.^117^

**FIGURE 7:**
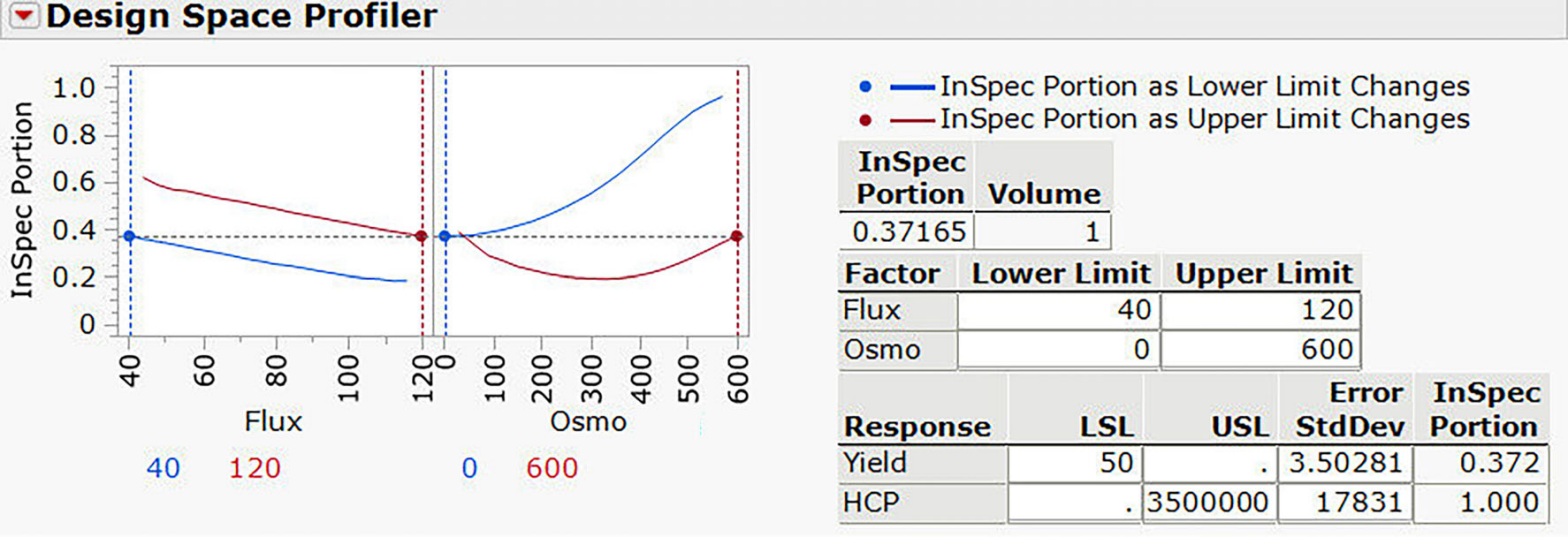
Example of design space profiler for clarification.

After reviewing the Prediction Profiler, researchers can define the design space using a Design Space Profiler for the target CQAs. For instance, if the target levels for the CQAs are a product yield above 50% and HCP below 35,000 ppm, the range of process parameters can be established and validated statistically. Figure 7.^117^ shows the example of design space profiler from JMP software.

Based on these results, the researcher may choose to perform a verification run at a laboratory scale for an evaluation of the process parameters defined by the DoE; the range of parameters could be changed from the normal operating range (NOR) to proven acceptable range (PAR).

#### 5.4.3 Process development—Scale‐up, tech transfer, gap analysis

**FIGURE 8:**
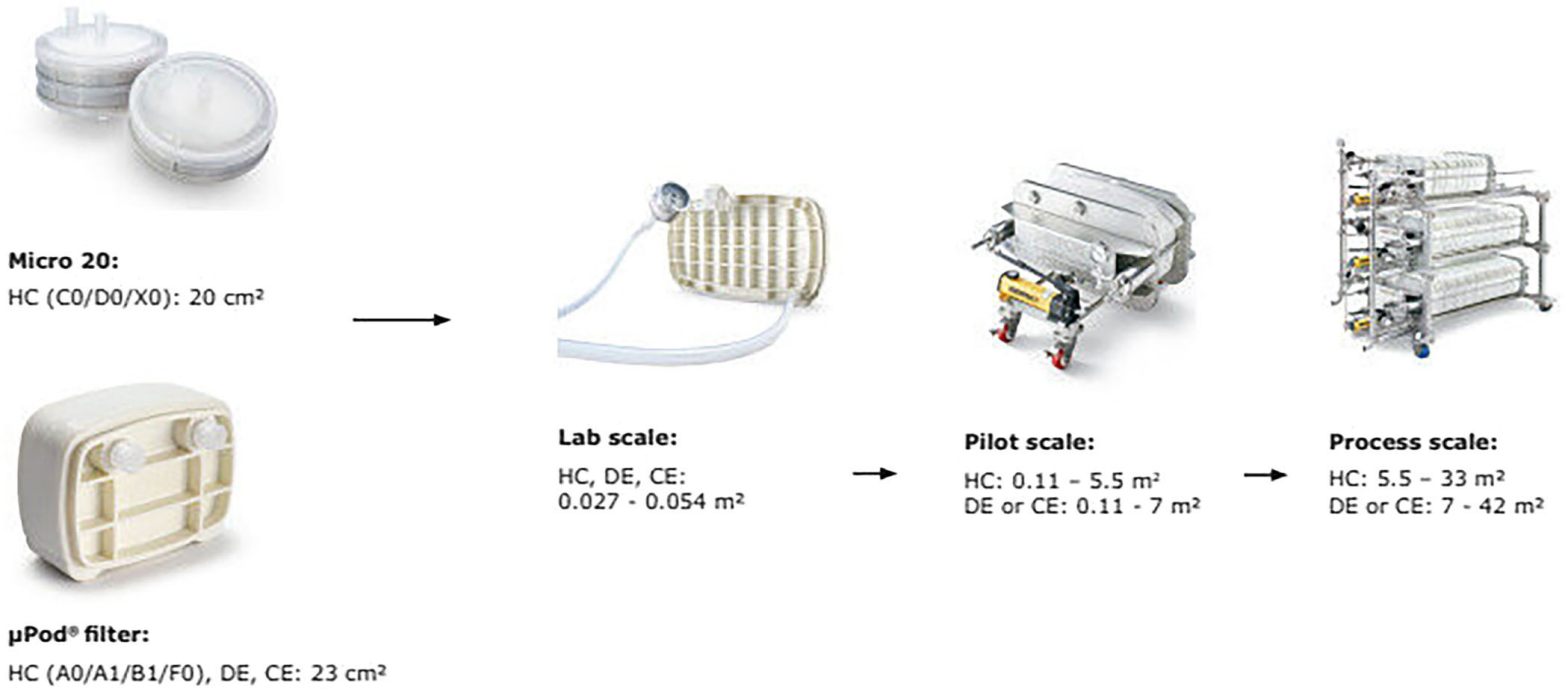
Example of clarification system by scale from a consumable supplier.^116^

From the design space study, verification runs may comprise from 3 to 5 runs for proving accuracy for statistical means and standard deviation. The verification runs will be proven to be normal, and these runs will have comparability with a proven design by DoE. These verification runs are considered to be representative of lab scale. For the scale‐up to manufacturing, such as over 2000 L scale, the intermediate testing of the process at a pilot scale is recommended for a stable scale‐up. Table 11 and Figure 8, ^116^ show an example of filter loading requirements for various cell culture harvest conditions and two‐stage depth filter media configurations for the required application. Many depth filter consumable suppliers provide guidance documents to assist in depth filter selection.

**TABLE 11:** Example of selection for depth filters by consumable supplier (Life Science business of Merck KGaA, Darmstadt, Germany).^116^

Cell density		Low (≤10 E 7 cells/ mL)			
		▼		▼	
Cell viability		Low viability		High viability	
		▼	▼	▼	▼
Depth filter train		Two‐stage		Two‐stage	
Millistak + ® media grade		HC Pro D0SP/X0SP	D0HC/X0HC	HC Pro D0SP/X0SP	D0HC/X0HC
Approx. expected filter loading capacity (L/m^2^)		D0SP: 240 X0SP: 120	D0HC: 120 X0HC: 120	D0SP: 120 X0SP: 250	D0HC: 120 X0HC: 250
Scale	1 L	Micro‐20 or μPod® device format			
	10 L	Lab‐scale pod (LSP) device format			
	100 L	Process‐scale pod (PSP) device format			
	1000 L				
	2000 L				
	2000 L+	See centrifuge			

Cell density		High (>10 E 7 cells/mL)			
		▼		▼	
Cell viability		Low viability		High viability	
		▼		▼	
Depth filter train		Two‐stage		Two‐stage	
Millistak + ® media grade		HC Pro C0SP/X0SP†	C0HC/X0HC	HC Pro D0SP/X0SP	D0HC/X0HC
Approx. expected filter loading capacity (L/m^2^)		C0SP: 100 X0SP: 120	C0HC: 50 X0HC: 120	D0SP: 140 X0SP: 150	D0HC: 70 X0HC: 150
Scale	1 L	Micro‐20 device format			
	10 L	Lab‐scale pod (LSP) device format			
	100 L	Process‐scale pod (PSP) device format			
	1000 L				
	2000 L				
	2000 L+	See centrifuge			

Small‐scale depth filter devices for lab‐scale filter screening and sizing operations are available. These small scale devices (20–25 cm^2^), are generally suitable for handling HCCF volumes from under 0.5 up to 1 L, depending on the specific application requirements. However, this area is considered to be insufficient for pilot‐scale experiments, which typically range from 10 to 100 L. The appropriate pilot scale device filter area can vary significantly between different suppliers, with typical filter areas ranging between 0.11 and 0.55 m^2^. These sizes are appropriate for 5 to 100 L scales, often considered pilot scale in certain contexts.

For larger scales, ranging from 100 to 2000 L, which span from pilot to process scales, hardware systems comprising multiple depth filter devices with filtration areas greater than 1.1 m^2^ are required. In order to accommodate the increased volume, these devices may be configured to provide filter areas of over 10–30 m^2^.

In addition to providing data to support the scale up of depth filtration devices, scale‐up studies may also be used to support comparability studies. These studies are crucial for ensuring that CQAs are maintained at various scales, from lab‐scale through to manufacturing scale, and remain consistent with no significant deviations. Verification processes such as these involve conducting preliminary trials at the lab scale, utilizing established parameter ranges. These parameters are then applied and evaluated at the pilot scale, and the evaluation findings are compared against those from the lab scale to ensure consistency. The same parameters are subsequently applied and assessed at the manufacturing scale, with statistical methods such as *t*‐test, Paired *t*‐test, ANOVA, and Sigma Quality Level theory employed to verify that there are no discrepancies across scales.

Despite these measures, the lack of significant differences in CQAs across scales does not automatically resolve all potential quality concerns. Effective quality control and strategic management require that researchers and manufacturers perform a comprehensive gap analysis from lab scale to manufacturing scale. This analysis helps to identify and address the myriad conditions that can vary across scales, ensuring that the scale‐up process supports the maintenance of product quality and compliance with regulatory standards.

This gap analysis also entails risk assessment. Therefore, researchers and manufacturers will employ methods such as FMEA, FTA, or HAZOP for risk assessment. For example, the calculation of the RPN using FMEA will be revisited for this gap analysis.

The gap analysis can be conducted before the technology transfer to manufacturing (Tech Transfer). As part of the tech transfer, numerous documents will be reviewed. Among these, the report of the process development study is crucial for implementation in the manufacturing plant. Target scores from this gap analysis may vary from company to company or product to product. Therefore, it is recommended that the method of risk assessment be specifically tailored to the manufacturer's own unique situation and manufacturing environment requirements.

## 6 CONCLUSION

In biopharmaceutical manufacturing processes, the clarification process has historically been considered a part of the upstream harvest process, and any process‐related impurities from this process step were considered to have minimal impact on Critical CQAs. Consequently, the importance of the clarification process has been undervalued. In this review article, we evaluate the significance of clarification from a QbD perspective. We describe the potential of various clarification process parameters such as flux, osmolality, conductivity, and pH buffer conditions to be critical process parameters. An evaluation of these parameters also supports the possibility of controlling impurities defined as CQAs. In addition, we describe methods of using DoE to set parameters for these studies. Beyond process development, we describe risk assessment methods that allow for improved quality control throughout the clarification process. The increasing utilization of depth filtration products as a downstream unit operation for haze removal and soluble impurity clearance further increases the relative importance of clarification from a QbD perspective.

An increased emphasis on quality control methods not only aligns with modern regulatory expectations but also significantly enhances the reliability of biopharmaceutical manufacturing. By applying rigorous QbD standards to the clarification process, it helps to drive the biopharmaceutical industry to higher safety and quality standards. This proactive approach toward improving the quality and robustness of biopharmaceutical manufacturing processes helps advance the forefront of pharmaceutical innovation and patient care.

## AUTHOR CONTRIBUTIONS

**Kyeong‐won Yeop:** Writing—original draft; conceptualization; writing—review and editing; project administration; data curation; methodology; formal analysis. **Hyun‐ju Nam:** Writing—original draft; writing—review and editing; visualization; data curation; formal analysis. **Chang‐jae Shim:** Writing—original draft; writing—review and editing; visualization. **So‐mi Yang:** Writing—original draft; visualization. **Hyo‐won Kim:** Writing—original draft; writing—review and editing. **Cheon Ik Park:** Funding acquisition; supervision; **Subhasis Banerjee:** Writing—review and editing; supervision; visualization. **Yanglin Mok:** Funding acquisition; supervision.

## CONFLICT OF INTEREST STATEMENT

The authors declare that there is no conflict of interest that could be perceived as prejudicing the impartiality of the research reported.

## Data Availability

Data sharing not applicable to this article as no datasets were generated or analysed during the current study.
